# First report of a *bla*_NDM_-producing extensively drug resistant *Klebsiella pneumoniae* ST437 in Italy

**DOI:** 10.3389/fcimb.2024.1426817

**Published:** 2024-09-11

**Authors:** Sofia Chiatamone Ranieri, Vittoria Fabbrizi, Ada Maria D’ Amario, Maria Giuseppina Frascella, Valeria Di Biase, Cinzia Di Francesco, Stefania Di Sante, Luigino De Berardis, Massimo De Martinis, Massimo Partenza, Alexandra Chiaverini, Gabriella Centorotola, Cesare Cammà, Francesco Pomilio, Alessandra Cornacchia

**Affiliations:** ^1^ Operative Unit of Clinical Pathology and Microbiology, Department of Services, “G. Mazzini” Hospital, ASL of Teramo, Teramo, Italy; ^2^ Infectious Disease Unit, “G. Mazzini” Hospital, ASL of Teramo, Teramo, Italy; ^3^ Clinical Risk Management and Medico-Legal Unit, “G. Mazzini” Hospital, ASL of Teramo, Teramo, Italy; ^4^ General Internal Medicine Unit, “Maria SS. dello Splendore” Hospital, Giulianova, ASL of Teramo, Teramo, Italy; ^5^ Department of Life, Health and Environmental Sciences, University of L’Aquila, L’Aquila, Italy; ^6^ Long-Term Care Unit, “G. Mazzini” Hospital, ASL of Teramo, Teramo, Italy; ^7^ Orthopedics and Trauma Unit, “Maria SS. dello Splendore” Hospital, Giulianova, ASL of Teramo, Teramo, Italy; ^8^ Istituto Zooprofilattico Sperimentale dell’Abruzzo e del Molise G. Caporale, Teramo, Italy

**Keywords:** carbapenemase-producing pathogen, New Delhi metallo-β-lactamase, *Klebsiella pneumoniae*, ceftazidime/avibactam, whole genome sequencing (WGS)

## Abstract

Carbapenemase-producing *Klebsiella pneumoniae* strains (CP-Kps) have recently been observed to spread rapidly worldwide. New Delhi metallo-β-lactamase (NDM) producing clones of *Klebsiella pneumoniae (K. pneumoniae)* cause a significant healthcare burden, particularly in Indian sub-continent, where this clone is circulating widely. However, in Italy, data on the incidence of these new clones is limited, and an ST437 NDM-producing *K. pneumoniae* strain has not been reported to date. A sacral ulcer infection caused by a *K. pneumoniae* strain was identified in an 85-year-old Italian male patient with several comorbidities. Antimicrobial susceptibility testing revealed an extensive resistance to a wide range of antimicrobials, including novel agents such as cefiderocol and ceftazidime/avibactam. Genomic analysis identified the pathogen as an ST437 *K. pneumoniae* strain harboring *bla*_NDM-5_, *bla*_OXA-232_ and *bla*_CTX-M-15_ genes. Following the identification of this first case, several infection control measures were implemented in healthcare settings, including direct precautions and reinforcement of standard cross-transmission control measures. The emergence of pathogenic microbial clones carrying new genetic determinants, particularly in a little city, requires prompt diagnosis and therapeutic protocols. An effective infection control system for the early detection and/or control of the transmission of NDM-producing *Enterobacteriaceae* is also needed. Further investigations are required to better understand the potential transmission routes and evolution of these clones.

## 1 Introduction

Carbapenemase-producing *Klebsiella pneumoniae* strains (CP-Kps) have spread rapidly around the world (Guo et al., 2023). The European Antimicrobial Resistance Surveillance Network (EARS-Net) of the European Centre for Disease Prevention and Control (ECDC) have recently reported a rate of 24.9% for carbapenem-resistant *Klebsiella pneumoniae (Kp)* isolates in Italy (European Centre for Disease Prevention and Control, 2023). New Delhi metallo-β-lactamase (NDM) is able to hydrolyse almost all β-lactams, including carbapenems. Since its 2008 discovery from a *Kp* strain isolated from a patient repatriated to Sweden after hospitalization in New Delhi, NDM-positive strains have been causing healthcare-associated outbreaks worldwide (Wu et al., 2019).

In Italy, the first outbreak of NDM-producing *Kp* (November 2018-May 2019) was documented in the northwestern area of Tuscany. This outbreak, involving nine different hospitals and 350 patients, was mostly caused by an NDM-1-producing *Kp* clone of ST147 (European Center for Disease Prevention and Control, 2019; Di Pilato et al., 2022).

Here, we report the identification of a *bla*_NDM-5_ and *bla*_OXA-232_ producing *Kp* clone of ST437, isolated for the first time from a patient hospitalized in a healthcare setting in Abruzzo region, Southern Italy.

## 2 Case presentation

In April 2023, an 85-year-old Italian male patient was admitted to the hospital with a proximal femoral fracture, and underwent emergency surgery. The patient had comorbidities, particularly senile dementia associated with Parkinsonism, arterial hypertension, and a second stage sacral pressure ulcer due to reduced mobility.

On the 9^th^ day after surgery, the patient developed fever and hypotension due to elevated lactate and procalcitonin levels (73 ng/ml). Blood and urine samples were collected, and empirical antibiotic treatment with meropenem 500 mg Q12H IV and vancomycin 1000 mg Q24H IV (renal impairment dosing) was initiated. On the 48^th^ hour, a suspected *Kp* strain was detected in both blood and urinary cultures. The isolate (*Kp*1) was identified as *Klebsiella pneumoniae* by matrix assisted laser desorption ionization-time of flight (MALDI-TOF) mass spectrometry using Vitek MS (Biomérieux, Marcy l’Étoile, France). Carbapenemase genes were screened using the Amplex Eazyplex SuperBug CRE test (Amplex Diagnostics, Gars am Inn, Germany) based on the loop-mediated isothermal amplification (LAMP) method. The *bla*_KPC_ and *bla*_CTX-M1_ genes were also detected).

The antimicrobial susceptibility test (AST) was performed using the MicroScan WalkAway system DxM 1096 (Beckman Coulter), and the results were interpreted according to the guidelines of the European Committee on Antimicrobial Susceptibility Testing (EUCAST, 2023, version 13.1). Since the breakpoint of tigecycline against *Kp* has not been made available by EUCAST, the EUCAST breakpoint for *E.* coli was used. Cefiderocol (FDC) susceptibility was tested using Minimum Inhibitory Concentration (MIC) Test Strip (Liofilchem, Roseto degli Abruzzi, Italy), and interpreted according to EUCAST clinical breakpoints. FDC is a novel injectable siderophore cephalosporin that is active against most carbapenem-resistant *Enterobacteriaceae* and is used for the treatment of complicated urinary tract infections, hospital-acquired bacterial pneumonia, and ventilator-associated bacterial pneumonia (Wu et al., 2020).

The results indicated that a modification to the antibiotic therapy may be required (**Table 1**). Hence, ceftazidime/avibactam (CZA) 2.5 g Q8H IV treatment was initiated against carbapenem-resistant *Kp* (Tumbarello et al., 2019). The patient’s clinical condition improved rapidly after microbiological eradication (negative blood and urine cultures).

**Table 1:** Minimum inhibitory concentration (MIC) and susceptibility results of *Kp*1 and *Kp*2 strains.

	*Kp*1 ST307		*Kp*2 ST437	
	MIC (mg/L)	Susceptibility	MIC (mg/L)	Susceptibility
AMI	<=8	S	>16	R
AMC	>32	R	>32	R
AMP	>8	R	>8	R
AZT	>16	R	>16	R
FEP	>8	R	>8	R
CTX	>32	R	>32	R
CAZ	>32	R	>32	R
CZA	<=2	S	>8	R
CZT	>4	R	>4	R
FUR	>8	R	>8	R
CIP	>1	R	>1	R
COL	<=2	S	<=2	S
FDC	NA	NA	8	R
ETP	>1	R	>1	R
ERA	NA	NA	<=0.5	S
GEN	<=2	S	>4	R
LVX	>1	R	>1	R
MEM	>8	R	>32	R
MEM/VAB	NA	NA	>16	R
TZP	>16	R	>16	R
TIC	>16	R	>16	R
TGC	<=0.5	S	<=0.5	S
TOB	<=2	S	>4	R
SXT	>4/76	R	>4/76	R

NA, not applicable.

**Figure 1:**
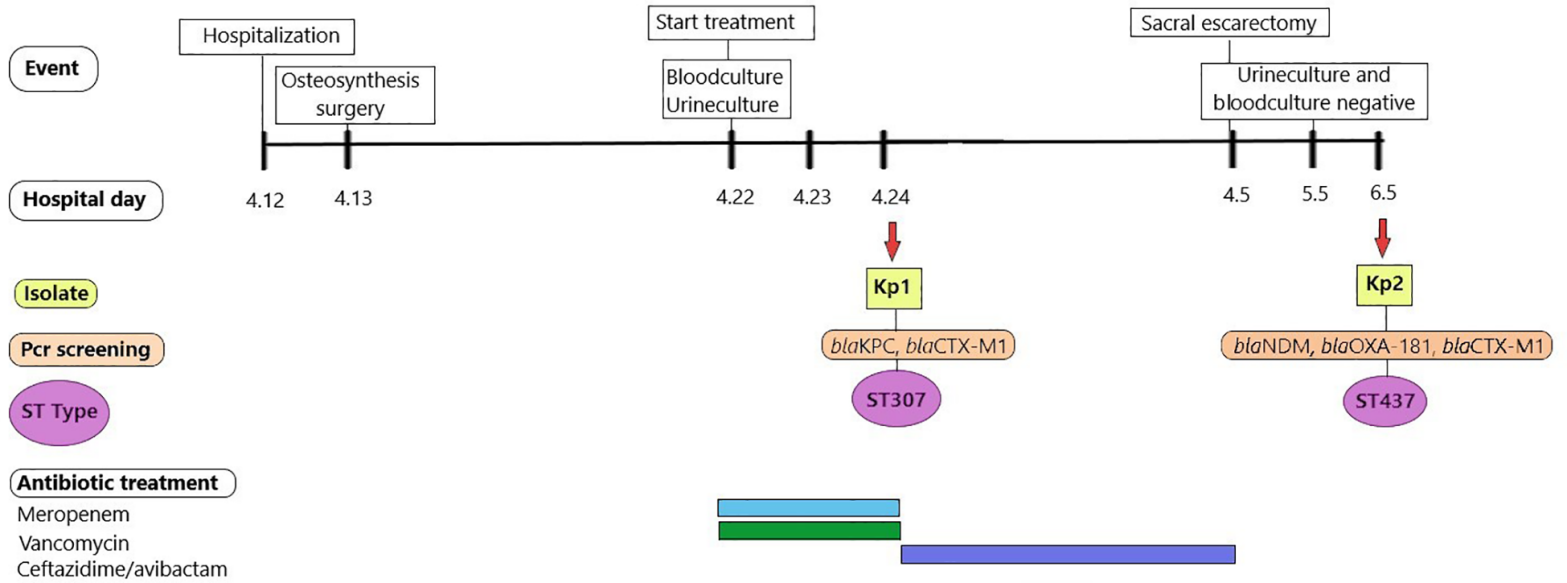
Medical history of the patient. Different colored bars in antibiotics treatment represent different antibiotics: light blue bar (Meropenem), green bar (Vancomycin), purple bar (Ceftazidime-avibactam).

On the 12^th^ day of antibiotic therapy (2 days of meropenem and 10 days of CZA), the patient underwent surgical toileting due to clinical worsening of the sacral ulcer, and vacuum-assisted closure therapy was initiated with gradual improvement of the lesion. Tissue sampling for microbiological examination was performed, and a different *Kp* strain (*Kp*2) was isolated and identified from the culture (**Table 1**). Screening of carbapenemase genes in the *Kp2* strain revealed the presence of *bla*_NDM_, *bla*_OXA-181_ and *bla*_CTX-M1_ genes. Considering the microbiological eradication of *Kp1*, CZA was interrupted and no indication for further antibiotic therapy was given, considering *Kp2* to be a colonizing strain of the sacral ulcer. The medical histories of the patients and laboratory analyses of *Kp* strains are outlined in **Figure 1**.

Both *Kp*1 and *Kp*2 strains were subjected to whole-genome sequencing (WGS). DNA was extracted according to the protocol described by Cornacchia et al. (2022), and WGS was performed using the Illumina platform (Cornacchia et al., 2022). An in-house pipeline was used for the WGS data analysis. The genome assembly quality check was assessed according to Hennart et al. (2022) (n. contigs < 1,000, total length ranged from 4.5 to 6.5 Mbp, GC% < 59%). Species confirmation and K/O locus determination were performed in Kleborate (Lam et al., 2021) hosted on the PathogenWatch platform (Argimón et al., 2021); meanwhile, sequence type (ST) was calculated *in silico* according to the multilocus sequence typing (MLST) scheme hosted on the Pasteur sdb platform (Diancourt et al., 2005 and Brisse et al., 2009). Chromosomal and acquired resistance genes and outer membrane porin alterations were detected by querying the ResFinder 4.4.2 database (v. 12th December 2023) (Florensa et al., 2022).

## 3 Results

WGS results confirmed that both strains belonged to species *Klebsiella pneumoniae*. MLST analysis revealed that *Kp*1 isolated from the blood and urine was a clone of ST307-CG307, and *Kp*2 from the sacral ulcer was a clone of ST437-CG10268. Capsular type (KL) and O locus (OL) analyses revealed that *Kp*1 belonged to KL102, OLO1/O2v2, and the capsular polysaccharide type was wzi173, while *Kp*2 belonged to KL52, OL101, and the capsular polysaccharide type was wzi50.

We focused on the *Kp*2 strain due to its phenotypic antimicrobial resistance and the presence of *bla*_NDM_ gene, which had never been identified in the healthcare system of our province before. The AST (**Table 1**) showed that *Kp*2 was extensively resistant, in particular to the combinations of cephalosporins/β-lactamase inhibitors such as CZA or carbapenem/β-lactamase inhibitor meropenem/vaborbactam (MEM/VAB). Resistance was also observed for FDC. *Kp*2 was susceptible only to tetracyclines (eravacycline (MIC < 0.5 μg/ml) and tigecycline (MIC < 0.5 μg/ml)) and polymyxins (colistin (MIC <=2 μg/ml)). Based on the resistance phenotype, this strain is classified as extensively drug resistant (XDR) (Magiorakos et al., 2012).

Carbapenem resistance genes *bla*_NDM-5_ and *bla*_OXA-232_ and Extended Spectrum β-Lactamase (ESBL) *bla*_CTX-M-15_ genes were identified through querying the ResFinder 4.4.2 database. The other penicillin resistance genes identified were *bla*_TEM-1D_ and *bla*_SHV-11_. Finally, trimethoprim (*dfr*A12), sulfonamide (*sul*1), and aminoglycoside (*aac*(6’)-Ib, *aad*A2, *rmt*B, *rmt*F) resistance genes were also identified. The GyrA-83I and ParC-80I mutations were also detected.

The presence of ompK36 with a glycine-aspartate (GD) L3 insertion was detected. The strain harbored several plasmid replicons (100% identity), including Inc-type (IncFII(K), IncFII, IncFIB(K), IncR) and Col-type (ColKP3, Col(BS512)) replicons. The ST437 strain was shown to have yersiniabactin ybt 14 located within the integrative conjugative element ICEKp5 to specifically overcome host-mediated iron limitation.

## 4 Discussion

To the best of our knowledge, this is the first report of the identification of an ST437 *bla*_NDM-5_ producing *Kp* in the Abruzzo region of Italy. The ST437 *Kp* is a global epidemic clone, previously reported in North and South America (Wang et al., 2013; Tijet et al., 2014; Aires et al., 2020), Europe (Emeraud et al., 2022; Benulič et al., 2020; Palmieri et al., 2020; Fuster et al., 2020; Marti et al., 2017) and Asia (Zhu et al., 2018; Weng et al., 2020; Sahoo et al., 2023).

The ST437 clone is often associated with β-lactamase-producing genes, mainly *bla*_KPC-2_ and *bla*_CTX-M-15_, and more recently with *bla*_OXA-like_ (*bla*_OXA-48_ and *bla*_OXA-245_) or *bla*_NDM_-_like_ (specifically *bla*_NDM-1_, *bla*_NDM-7_ and *bla*_NDM-23_) genes. ST437 *bla*_KPC-2_ and *bla*_CTX-M-15_ expression was previously reported in isolates from Brazil (Andrade et al., 2011; Palmeiro et al., 2019) and *bla*_KPC-2_ and *bla*_OXA-1_ in Ontario, Canada (Tijet et al., 2014).

In Europe, several studies have reported the presence of ST437 clones as well. For instance, *bla*_OXA-245_ producing *Kp* (Oteo et al., 2013), *bla*_NDM-7_ producing *Kp* (Seara et al., 2015) and *bla*_OXA-48_ and *bla*_NDM-1_ *Kp* were identified in Spain (Fuster et al., 2020).

Benulič et al. described the population of carbapenem-resistant *Kp* detected during an outbreak occurred in Slovenia between 2014–2017, including *bla*_OXA-48_ and/or *bla*_NDM-1_ ST437 strains. Palmieri et al. (2020) reported the presence of ST437, *bla*_OXA-48_ and *bla*_NDM-1_ in clinical *Kp* isolates from Serbia. In the same year, Weng et al. described for the first time a *bla*_OXA-232_ producing ST437 *Kp* strain in China. The *bla*_OXA-232_ producing strains are frequently associated with travel to India.

In 2023, *bla*_NDM-5_ ST437 *Kp* was isolated from a river in eastern India (Sahoo et al., 2023).

In Italy, the ST437 *Kp* clone is rarely detected, with previous reports in two hospitals in Padua between June 2009 and December 2011, which highlighted the presence of *bla*_KPC-2_ gene.

The ST437 *Kp2* clone detected in our hospital was susceptible to colistin and tigecycline, and showed elevated MIC values of all carbapenems, cephalosporins and also novel β-lactamase inhibitor combinations (BLICs) tested. The *bla*_NDM-5_ carbapenemase identified in the ST437 *Kp* explains all β-lactam resistance observed, with the exception of that to aztreonam, which is not hydrolyzed by metallo-β-lactamases. However, this strain also carries *bla*_CTX-M-15_ as well as another class D carbapenemase, *bla*_OXA-232_, which may contribute to aztreonam resistance (de Man and Limbago, 2016). In this context, the use of avibactam protects aztreonam from hydrolysis, making it effective against NDM (Tamma et al., 2023).

High MICs for tobramycin, amikacin, and gentamicin were also observed, most likely due to the presence of *rmtF*. Fluoroquinolone resistance is also likely affected by the presence of *GyrA-83I* and *ParC-80I* mutations. The ST437 *Kp* isolate, in addition to *bla*_NDM-5_ and *bla*_OXA-232,_ carried several mutated porin genes in *ompK36* and *ompK37* which are linked to resistance to cephalosporins and carbapenems. The presence of carbapenems and the co-occurrence of other resistance determinants (*bla*_NDM-5_, *bla*_CTX-M_, *bla*_SHV_, and *bla*_TEM_), virulence factors (*ybt14*), and the capsular serotype (KL52) further highlights its high pathogenic potential.

Among the *bla*_NDM_ variants, *bla*_NDM-5_ was first reported in the UK in 2011 and has gained significant attention owing to its enhanced resistance to carbapenems and broad-spectrum cephalosporins (Li et al., 2018).

Infections caused by bla_NDM-5_-carrying CP-Kps are associated with high rates of morbidity, mortality, and transmission due to the dissemination ability of NDM-producing *Kp* strains (Paczosa and Mecsas, 2016; Jia et al., 2022).

In this study, the acquisition of bla_NDM-5_-producing *Kp* by the patient could not be explained by direct contact with a colonized patient. However, the potential involvement of undetected and asymptomatically-colonized patients in transmission routes cannot be excluded. The selective pressure of CZA treatment may have also favored the emergence of this NDM-producing *Kp* strain.

The case described here had no proven connection with India, Pakistan, the Middle East, the Balkans, Spain in Europe, Brazil, or North America. Therefore, the route through which NDM-producing *Kp* was introduced into the hospital is unknown. According to European Centre for Disease Prevention and Control (2011), patients with no direct link to countries where this clone is circulating are defined as autochthonous cases, indicating an unknown reservoir of NDM.

Two STs clones with similar virulence potentials and resistance profiles were also identified. Hence, a single dominant clone is not the only one responsible for severe infections, causing repercussions on prognosis and therapeutic treatment (Venturini et al., 2022). Although the number of bacterial isolates belonging to the same species analyzed per sample is determined by response times, increases in testing capacity and improved diagnostic protocols are needed to improve preciseness in the identification procedure. The ST437 *Kp*2 clone was considered by the infectious disease clinician to be a colonizing strain, in fact after surgical toilet and vacuum-assisted suture therapy the lesion gradually improved and no local or systemic signs of active infection occurred. For these reasons the patient did not receive further antibiotic treatment. Nevertheless, antibiotic pressure plays a decisive role in modifying the susceptibility of microorganisms to various antibiotics (Shields et al., 2017).

Approximately one-third of deaths caused by AMR in Europe occur in Italy (https://www.ecdc.europa.eu/en/publications-data/antimicrobial-resistance-surveillance-europe-2022-2020-data). Italy is one of the countries with a high rate of antibiotic resistance, and where antibiotics are used excessively and often inappropriately, although there are signs of improvement, as reported in the last Ears-NET report (carbapenem-resistant isolates percentage from 26.7% to 24.9%).

Southern regions of Italy have critically higher levels of carbapenem-resistant *Kp* strains than the northern parts. In Southern Italy, Abruzzo, Apulia and Campania are the regions with the highest levels of prevalence of antimicrobial use (around 45%) (Medicines use in Italy National Report Year 2022 Published on: 01 December 2023).

Pérez-Galera et al. (2023) described several risk factors, which may contribute favoring Carbapenem-resistant *Enterobacteriaceae* (CRE) infections. These factors include hospitalization in the last three months, previous colonization/infection by CRE, chronic heart failure, dementia, chronic renal failure, central venous and urinary catheters, dialysis, and previous use of antibiotics. Moreover, significant comorbidities and invasive surgeries play a key role in the deterioration of the immune status conditions of hospitalized patients (European Centre for Disease Prevention and Control, 2011; Zuo et al., 2021). Most of the previously described risk factors were identified in the present case.

After the first case of NDM-producing ST437 *Kp*, several infection control measures were implemented in our healthcare setting, including contact precautions in individual rooms, reinforcement of standard cross-transmission control measures, cleaning procedures, and active surveillance cultures, together with retraining courses for operators. All these measures were adopted to raise awareness of this issue and avoid the spread of this strain associated with high mobility and mortality in our patients (World Health Organization, 2016 and 2017). All control measures activated and surveillance activities (culture swabs combined with molecular methods) were successful, indeed only one positive patient was identified. The patient positive for the presence of the ST437 *Kp* strain was a 92 years old woman, bedridden, with a long hospital stay and a bladder catheter, therefore with many risk factors for colonization. After the first detection of this strain in the hospital, other rare and sporadic findings of ST437 *Kp* strain were recorded (6 cases of urinary infections) in the following 7 months. In all cases, infection control measures effectively prevented the spread of the strain among patients. Heterogeneity in coverage across the wards in the hospital and the use of culture and not molecular methods for the identification of carbapenem resistant strains in screening practice, limits the knowledge of the real spread of the ST437 *Kp* strain in our healthcare setting.

The effectiveness of infection control interventions for the early detection and/or control of the transmission of NDM-producing *Enterobacteriaceae* has not been proven yet. However, an ECDC risk assessment report demonstrated the effectiveness of active surveillance and control measures, including a dedicated team for all suspected and CPE-positive patients (European Centre for Disease Prevention and Control, 2011). The emergence of clones carrying new genetic determinants, especially in local areas and small regions such as the Abruzzo region, requires prompt diagnosis and therapeutic protocols, as well as further investigations to better understand the potential transmission routes and evolution of these clones. This case report is the snapshot of a restricted geographic area in a limited period of time and its origin is still unknown. For this reason, a more active AMR surveillance involving other hospitals in Abruzzo and the other Italian regions combined with next generation methods could potentially improve *Kp* infection treatment and patient outcomes. It is time for a more effective and widespread antimicrobial stewardship with a multidisciplinary approach to be implemented in hospitals and outpatients. These strategies should involve local, regional, and central government monitoring, auditing, and feedback.

## Data Availability

The datasets presented in this study can be found in online repositories. The names of the repository/repositories and accession number(s) can be found below: https://www.ncbi.nlm.nih.gov/genbank/, SAMN40624209 https://www.ncbi.nlm.nih.gov/genbank/, SAMN40624210.
